# Changes in Rhizosphere Soil Microorganisms and Metabolites during the Cultivation of *Fritillaria cirrhosa*

**DOI:** 10.3390/biology13050334

**Published:** 2024-05-11

**Authors:** Zhixiang Liu, Jizhe Ying, Chengcheng Liu

**Affiliations:** 1 Institute of Herbgenomics, Chengdu University of Traditional Chinese Medicine, Chengdu 611137, China; 2 College of Pharmacy, Hubei University of Chinese Medicine, Wuhan 430065, China; 17394662356@163.com

**Keywords:** *Fritillaria cirrhosa*, rhizosphere, metagenome, metabolome

## Abstract

**Simple Summary:**

During the cultivation of medicinal plants, there may be some changes in the rhizosphere microenvironment. We used metagenomic sequencing analysis combined with metabolomics to investigate the composition, changes, and interactions of the microbial community structure and metabolites in rhizosphere soil during *Fritillaria cirrhosa* cultivation. The results showed that with the increase in cultivation years, the beneficial bacteria in the rhizosphere soil decreased while the harmful bacteria increased, and the relative content of some soil nutrients significantly decreased. The research results will provide guidance for the cultivation of *Fritillaria cirrhosa*.

**Abstract:**

*Fritillaria cirrhosa* is an important cash crop, and its industrial development is being hampered by continuous cropping obstacles, but the composition and changes of rhizosphere soil microorganisms and metabolites in the cultivation process of *Fritillaria cirrhosa* have not been revealed. We used metagenomics sequencing to analyze the changes of the microbiome in rhizosphere soil during a three-year cultivation process, and combined it with LC-MS/MS to detect the changes of metabolites. Results indicate that during the cultivation of Fritillaria cirrhosa, the composition and structure of the rhizosphere soil microbial community changed significantly, especially regarding the relative abundance of some beneficial bacteria. The abundance of Bradyrhizobium decreased from 7.04% in the first year to about 5% in the second and third years; the relative abundance of Pseudomonas also decreased from 6.20% in the first year to 2.22% in the third year; and the relative abundance of Lysobacter decreased significantly from more than 4% in the first two years of cultivation to 1.01% in the third year of cultivation. However, the relative abundance of some harmful fungi has significantly increased, such as Botrytis, which increased significantly from less than 3% in the first two years to 7.93% in the third year, and Talaromyces fungi, which were almost non-existent in the first two years of cultivation, significantly increased to 3.43% in the third year of cultivation. The composition and structure of *Fritillaria cirrhosa* rhizosphere metabolites also changed significantly, the most important of which were carbohydrates represented by sucrose (48.00–9.36–10.07%) and some amino acid compounds related to continuous cropping obstacles. Co-occurrence analysis showed that there was a significant correlation between differential microorganisms and differential metabolites, but Procrustes analysis showed that the relationship between bacteria and metabolites was closer than that between fungi and metabolites. In general, in the process of *Fritillaria cirrhosa* cultivation, the beneficial bacteria in the rhizosphere decreased, the harmful bacteria increased, and the relative abundance of carbohydrate and amino acid compounds related to continuous cropping obstacles changed significantly. There is a significant correlation between microorganisms and metabolites, and the shaping of the *Fritillaria cirrhosa* rhizosphere’s microecology by bacteria is more relevant.

## 1. Introduction

*Fritillaria cirrhosa* (FC) is a perennial herbaceous plant belonging to the genus *Fritillaria* in the Liliaceae family [1]. FC serves as a primary botanical reservoir of Chuan Bei Mu, a traditional Chinese medicinal herb, showcasing noteworthy therapeutic efficacy in addressing diverse respiratory ailments including cough, sputum, pneumonia, bronchitis, and asthma, while also exhibiting promising potential as a pharmacological intervention for COVID-19 [2,3,4]. For thousands of years, Chuan Bei Mu has been a precious traditional Chinese medicine for moistening the lungs and relieving cough. It mainly suppresses the cough center rather than the respiratory center to suppress cough, and has excellent therapeutic effects on patients with chronic bronchitis complicated by emphysema and cough [5]. The alkaloid components in Chuan Bei Mu, such as Peimine, Peiminine, Sipeimine, and isoverticine, can effectively prolong the cough latency of mice and reduce the number of coughs [6,7]. The expectorant activity is another important activity of Sichuan Fritillaria. The total alkaloid components extracted from Sichuan Fritillaria have been proven to have expectorant effects [6]. In recent years, a large amount of the literature has reported that Chuan Bei Mu displays anti-tumor activity, including total alkaloids, triterpenes, and polysaccharides, which display anti-tumor activity against lung cancer cells, colon cancer cells, liver cancer cells, endometrial cancer cells, and ovarian cancer cells [2,8,9]. As a consequence of its restricted ecological, protracted reproductive cycle, and anthropogenic perturbations to its native habitat, FC has been designated and included in the IUCN Red List of Endangered Species [10]. Nevertheless, the global annual demand for Chuan Bei Mu surpasses 2000 tons, highlighting the insufficiency of relying solely on wild resources [10]. Fortunately, the challenges of cultivating FC through artificial means have been overcome, and the cultivation area of FC is expanding year by year [11]. However, due to its unique growth requirements and limitations of arable land, FC cultivation often involves continuous cropping, which exacerbates the issues of pests and diseases that arise from this practice [12,13,14].

In the cultivation process of medicinal plants hindered by the challenge of continuous cropping, many phenomena are displayed, including the inhibition of regular physiological activities, accumulation of allelopathic and autotoxic compounds, acidification, nutrient imbalances, and disrupted microbial community structure [15,16]. These cumulative factors ultimately result in suboptimal plant growth and impaired developmental progression. Simultaneously, the substantial reduction in the content of pharmaceutically active constituents within the medicinal parts significantly diminishes the quality of medicinal plants, severely constraining the health and sustainable development of the medicinal plant industry [17]. Abundant evidence suggests a close correlation between soil physicochemical properties, rhizosphere microbiota, and root exudates with the occurrence of continuous cropping obstacles [18,19,20]. For instance, the alteration of soil physicochemical properties serves as a primary causative factor in the occurrence of continuous cropping obstacles in *Panax ginseng* cultivation [21]. The occurrence of continuous cropping obstacles in *Morels* cultivation may be associated with variations in soil microbial community structure [22]. Moreover, continuous cropping also changes the components of *Sesamum indicum* root exudates [23].

With the advancement of omics technology, the integration of multiple omics approaches offers a comprehensive understanding to elucidate biological phenomena. Metagenomic analysis is similar to meta-analysis, which is defined as the analysis of analyses, and is used to gain analytical knowledge of a group of similar but different organisms [24]. In recent years, an increasing number of studies have employed this technology to explore the structure of rhizosphere soil microbial communities and elucidate their interactions with host plants [25,26]. Additionally, metabolomics is a new discipline that has emerged in recent years, focusing primarily on small molecules with molecular weights below 1000 Da, such as sugars, organic acids, lipids, amino acids, and so on. It can provide a complete and objective representation of the types of metabolites in the sample, and it can pay attention to the similarity, clustering, and differential changes between biological samples [27]. The ultra-high performance liquid chromatography-mass spectrometry (UPLC-MS) combined technology is widely used in the detection and quantification of plant rhizosphere soil metabolites. This technology has good separation and high sensitivity, and it can quickly and accurately analyze the metabolites contained in samples. Combined with multivariate statistical methods, differential metabolites are screened, and possible metabolic pathways and mechanisms of metabolites are predicted [27,28]. To the best of our knowledge, there has been limited advancement in elucidating the alterations in the rhizosphere microbial community’s structure and soil metabolites during the study of the FC cultivation process. In this work, we combined metagenomics and metabolomics to characterize the rhizosphere soil microorganisms and metabolomics during the growth and development of FC, providing new insights into the relationship between cultivation time, microbial communities, and their metabolites.

## 2. Materials and Methods

### 2.1. Obtaining FC Rhizosphere Soil Samples

The test site is located in Mianning County, Sichuan Province, China (28°55′ N, 102°10′ E). The base has a subtropical climate, with an altitude of up to 2800 m, an annual rainfall of 1095 mm, an average annual temperature of 13.8 °C, and an annual evaporation of 1857 mm. The soil in the experimental area is dark brown sandy loam. The cultivation of FC began in 2020. In the following three years, the seeds of FC were sown in March every year. All FCs were not transplanted during the cultivation period, and all FC management modes were the same. On 18 October 2022, the rhizosphere soil samples of FCs cultivated for one year, two years, and three years were collected via a zigzag sampling method, and they were numbered FC1, FC2, and FC3, with three replicates in each group. Each replicate was composed of 10 FC rhizosphere soil samples. The rhizosphere soil samples were transported to the laboratory on ice, and then they were frozen in liquid nitrogen and stored in a −80 °C refrigerator.

### 2.2. Soil Metabolome Analysis

Before the experiment begins, the compounds and reagents at the chromatographic level, such as methanol, formic acid, water, acetonitrile, and L-2-chlorophenylalanine, should be precooled at −20 °C. Accurately weigh 1000 milligrams of rhizosphere soil sample and place them in a 2 mL centrifuge tube. Add 1 mL of methanol/water solution (4:1; *v*/*v*) and freeze/digest at −10 °C, 50 Hz, for 6 min. Then, extract at a low temperature using ultrasound for 30 min (5 °C, 40 kHz). Store the sample solution at −20 °C for 30 min and centrifuge for 15 min (13,000× *g*, 4 °C). Transfer the supernatant to a new centrifuge tube and dry it with nitrogen gas. Store it in a −80 °C refrigerator. Before metabolite analysis, dissolve 100 micrograms of the sample again in acetonitrile/water (1:1, *v*/*v*), repeat the low-temperature ultrasonic centrifugation and centrifugation steps, and take the supernatant for analysis on the instrument. Take 20 µL of the supernatant from each sample and mix it as a quality control (QC) sample. In this experiment, an ultra-high performance liquid chromatography tandem time-of-flight mass spectrometry system (Thermo Scientific, Waltham, MA, USA) was used for analyzing rhizosphere metabolites. Separation conditions: chromatographic column: Hypsil Gold (100 mm × 2.1 mm, 1.9 µm); mobile phase A consists of 95% water + 5% acetonitrile (containing 0.1% formic acid), and mobile phase B consists of 47.5% acetonitrile + 47.5% isopropanol + 5% water (containing 0.1% formic acid); the flow rate is 0.40 mL/min, and the injection volume is 10 μL. The column temperature is 40 °C, and the sample is placed in a 4 °C automatic sampler throughout the analysis process. During the instrument analysis process, one QC sample is included in every three analysis samples to evaluate the stability of the entire detection process. The sample mass spectrometry signals were collected using positive and negative ion scanning modes, with a selection range of 125–1000 *m*/*z* for mass spectrometry scanning, a full scan resolution of 70,000, a high-resolution high-energy collision dissociation mass spectrometry (HCD MS/MS) scanning resolution of 17,500, and a capillary temperature of 320 V. The ESI source was set as follows: detection mode ESI+, spray voltage 3.5 kV, sheath gas flow 40 arb, and auxiliary gas flow 10 arb, as well as detection mode ESI−, spray voltage −3 kV, sheath gas flow 35 arb, and auxiliary gas flow 8 arb [29].

### 2.3. DNA Extraction, Library Construction, and Metagenomic Sequencing

DNA was extracted from FC rhizosphere soil according to the instructions of the DP336-02 TIAamp Soil DNA Kit (Omega Bio-Tek, Norcross, GA, USA), Nanodrop was used to detect DNA purity (absorbance ratio 260/280), 1% agarose gel electrophoresis was used to analyze DNA integrity, and qubit 2.0 was used to accurately quantify the DNA concentration. After passing the DNA sample detection, the library was constructed using the Covaris Ultrasonic Crusher. The library was diluted using Qubit 2.0, the insertion fragments of the library were detected using Agilent 2100, and the effective concentration of the library was accurately quantified using the Quantitative Polymerase Chain Reaction (Q-PCR) method. After passing library detection, the samples were double-ended sequenced using the Illumina Novaseq platform [29,30].

### 2.4. Statistical Analysis

Preprocess the raw data using KneadData software (0.7.4); Use FastQC (0.11.9) to test the rationality and effectiveness of quality control; use Kraken2 (2.0.7 beta) to identify the species contained in the classification sample; use Bracken 2.0 (default parameter) to estimate the abundance and species level of the classification results obtained from Kraken2 using the Bayesian algorithm; use HUMAnN 2 software (based on DIAMOND) and protein database (UniRef 90) for sequence comparison and annotation of functional genes; use the script human provided by HUMAnN_Renorm_Table to calculate the relative abundance of each protein in UniRef 90 based on its original abundance (parameter-units cpm, i.e., copy per million); according to the corresponding relationship between the ID of UniRef 90 and the Kyoto Encyclopedia of Genes and Genomes (KEGG) functional database (https://www.genome.jp/linkdb/, accessed on 18 October 2022), calculate the relative abundance of the functions and create abundance clustering heatmaps. For the metabolomic data collected offline, MassLynx V4.2 was used to perform peak extraction, peak alignment, and other data processing operations on the collected raw data using Progenesis QI 2.3 software. Based on Progenesis QI software, the online METLIN database was identified, and theoretical fragment identification was performed. The quality deviation was all within 100 ppm.

All figures and tables in this article are visualized by Wekemo Bioincloud (https://www.bioincloud.tech/, accessed on 18 October 2022) [31].

## 3. Results

### 3.1. The Alpha and Beta Diversity of Microbial Communities in FC Rhizosphere Soil Vary with Different Cultivation Years

**Figure 1:**
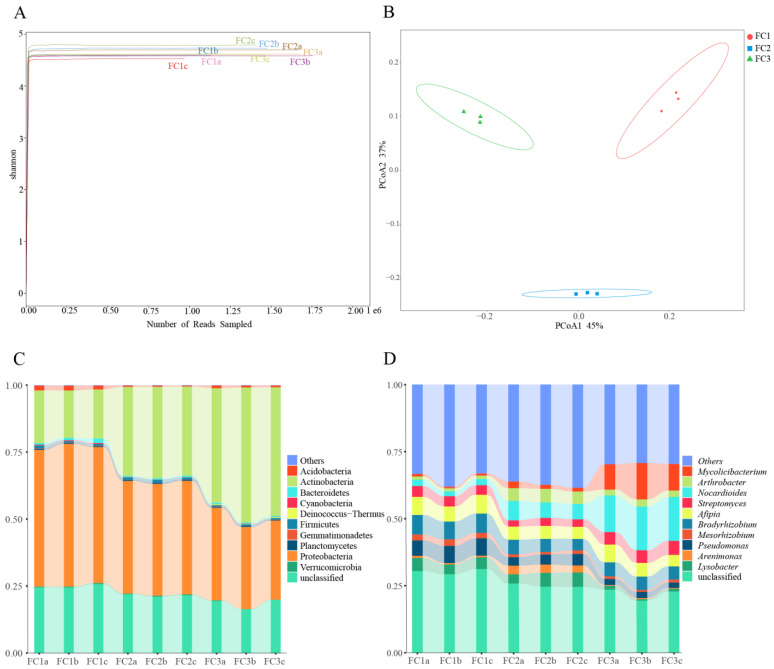
Diversity and composition of bacterial community in FC samples. (**A**) Shannon–Winner curve of bacterial community in FC samples. (**B**) Bacterial communities were based on Bray–Curtis PCoA analysis. (**C**) Composition of bacterial community at phylum level (TOP10). (**D**) Composition of bacterial community at genus level (TOP10).

**Figure 2:**
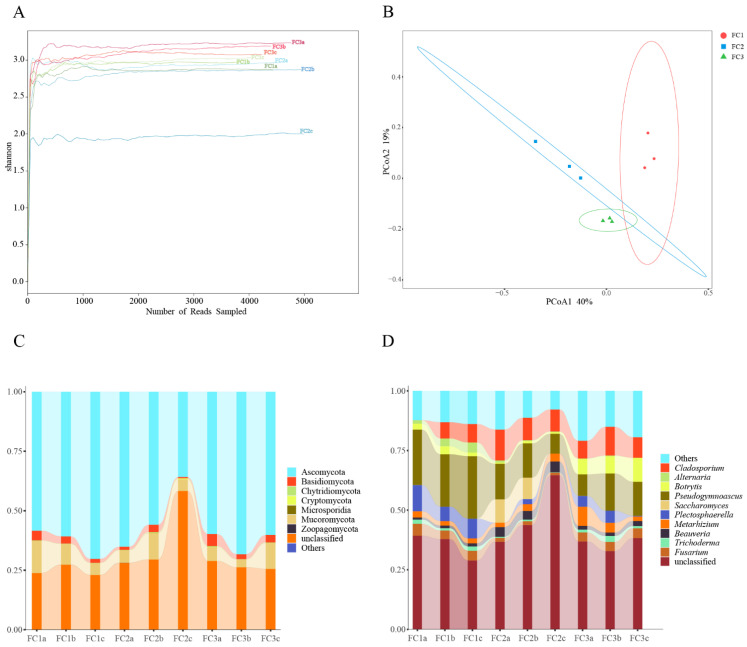
Diversity and composition of fungal communities in FC samples. (**A**) Shannon–Winner curve of fungal community in FC samples. (**B**) Fungal communities were based on Bray–Curtis PCoA analysis. (**C**) Composition of fungal community at phylum level (TOP10). (**D**) Composition of fungal community at genus level (TOP10).

In this study, a total of 207,163,760 raw reads were obtained from 9 samples following Illumina sequencing. Subsequently, a sum of 192,094,391 clean reads were acquired post quality control measures. Each sample’s sequencing data exhibit a Clean Q30 value surpassing 92%, thereby signifying the precision and accuracy achieved in our sequencing outcomes (Table 1). The rarefaction curves of bacterial and fungal species abundance in each sample gradually level off, indicating that the sequencing depth has adequately captured the diversity of species present in the samples (Supplementary Figure S1A). The Shannon–Winner curve provides a comprehensive view of the diversity patterns observed in different samples. After two or three years of cultivation, the rhizosphere soil microorganisms of FC Alpha (α) diversity were higher than FC1. This suggests that FC1 has a less diverse and complex bacterial community. On the other hand, after 3 years of cultivation in FC, the growth of rhizosphere soil fungi α diversity was higher than FC1 and FC2 (Figure 1A and Figure 2A).

Based on the principal co-ordinates analysis (PCoA) of the bacterial community, PCoA1 and PCoA2 account for 45% and 37% of the variation, respectively, and there was obvious separation among the three groups (Figure 1B). Combining the results of the analysis of similarities (ANOSIM) (R = 1, *p* = 0.004), it is evident that the differences between the groups are significantly greater than the differences within the groups (Supplementary Figure S1B). Similar results were obtained from the fungal community analysis (Figure 2B, Supplementary Figure S2B). These findings indicate that significant changes occur in both the bacterial and fungal communities in the rhizosphere soil of FC during the cultivation process.

### 3.2. Composition and Changes in Bacterial Communities

From the rhizosphere soil of FC, a total of 39 phyla, 67 classes, 147 orders, 324 families, 987 genera, and 4260 species of bacteria were discerned (Supplementary Table S1). Proteobacteria, with a relative abundance of 41.76%, stands as the most prevalent bacterial phylum, followed by Actinobacteria at 33.04%. Notably, these two phyla are the only ones exhibiting a relative abundance exceeding 1% (Supplementary Table S2). Among the top 10 bacteria in relative abundance, the relative abundance of Proteobacteria, Bacteroidetes, Verrucomicrobia, and Cyanobacteria decreased continuously with the cultivation of FC. The relative abundance of Actinobacteria was increasing. The relative abundances of Acidobacteria, Gemmatimonadetes, and Gemmatimonadetes decreased first and then increased. On the contrary, the relative abundances of Firmicutes and Plantomycetes increased first and then decreased (Figure 1C, Supplementary Table S2). Actinomycetia, Hypomicrobiales, and Nitrobacteraceae were the dominant class, order, and family species, respectively. In order to gain deeper insights into the fluctuations in bacterial abundance and composition during FC cultivation, an analysis of the bacterial communities in the rhizosphere soils of FC was conducted at the genus level. The relative abundances of *Nocardioides* and *Mycoicibacterium* showed an increasing trend, while the relative abundances of *Pseudomonas* and *Mesorhizobium* showed a decreasing trend (Figure 1D, Supplementary Table S3). Since metagenome sequencing analysis can accurately identify the species level, we explored the community composition of bacterial species level. The results indicate that the relative abundance of ten bacterial species exceeded 1%. Notably, *Nocardioides panacis* and *Mycolicibacterium gilvum* were significantly more abundant in the rhizosphere soil of the FC cultivar in the third year compared to other groups (Supplementary Figure S1C, Supplementary Table S4).

**Figure 3:**
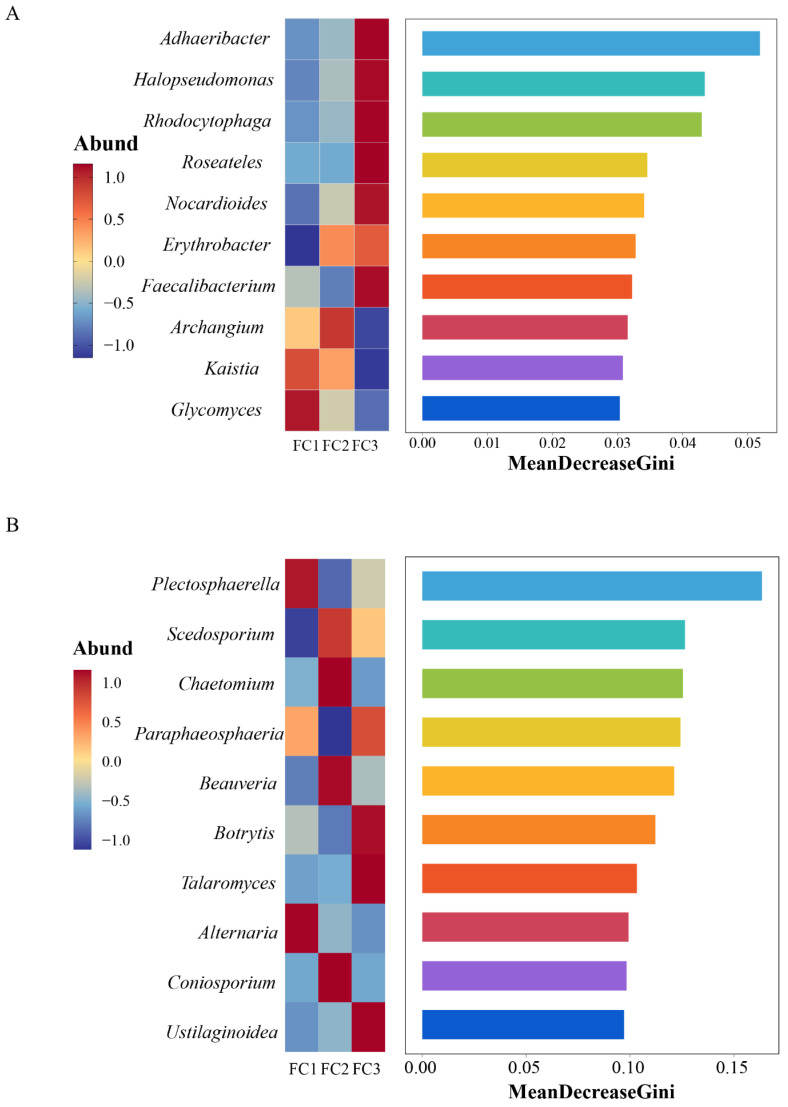
Random forest analysis was used to screen microorganisms of differential bacterial genera (**A**) and differential fungal genera (**B**).

Linear discriminant analysis Effect Size (LEfSe) was used to predict biomarkers of rhizosphere soil microorganisms during FC cultivation. A total of 21 biomarkers with Linear Discriminant Analysis (LDA) results of >4 were enriched (Supplementary Figure S1D). Among the 10 taxa of FC1, the bacteria of the Actinobacteria phylum and Actinomycetia class contributed the most. Six taxa were enriched in FC2, and the bacterial community of the order Micrococcales contributed the most. Proteobacteria phyla are the taxa with the largest contribution among FC5 biomarkers. Random forest was further used to find microbial genera with significant differences among groups (Figure 3A). The top 10 features with the highest contribution comprised *Adhaeribacter*, *Halopseudomonas*, *Rhodocytophaga*, *Roseateles*, *Nocardioides*, *Erythrobacter*, *Faecalibacterium*, *Archangium*, *Kaistia*, and *Glycomics*.

### 3.3. Composition and Changes in Fungal Communities

From the phylum to species level, 7, 26, 65, 129, 209, and 372 fungal communities were identified in FC rhizosphere soil, respectively (Supplementary Table S5). Only Ascomycota, Mucoromycota, and Basidiomycota had relative abundances exceeding 1%. And, their relative abundance accounted for 99.61% of the identified fungi (Figure 2C, Supplementary Table S6). Sordariomycetes, Hypocreales, and Pseudomicrobiotiaceae were the dominant fungi regarding class, order, and family, respectively. Similarly, we focused on the composition and changes of fungi at the genus level. The relative abundance of 11 fungal genera exceeded 1%; they were *Pseudonymnoascus* (16.44%), *Cladosporium* (8.27%), *Plectosphaerella* (4.16%), *Botrytis* (3.70%), *Fusarium* (3.18%), *Metarhizium* (3.17%), *Beauveria* (2.30%), *Saccharomyces* (2.09%), *Alternaria* (1.21%), *Trichoderma* (1.15%), and *Ilonectria* (1.03%) (Supplementary Table S7). After two or three years of cultivation, the relative abundance of *Pseudogymnoascus* and *Plectosphaerella* in the rhizosphere soil of FC was significantly lower than that of FC1. After three years of cultivation, the relative abundance of *Botrytis* and *Metarhizium* in the rhizosphere soil of FC significantly increased (Figure 2D). We also focused on the changes in fungal communities at the species level, and the two fungal species with the highest relative abundance were *Pseudonymnoascus verrucosus* and *Cladosporium cladosporioides*. Moreover, the relative abundance of the former showed a downward trend. In contrast, that of the latter established an upward trend (Supplementary Figure S2C, Supplementary Table S8).

A total of 11 biomarkers with LDA of >4 were selected via LEfSe analysis. At the genus level, *Plectospaerella* and *Alternaria* in FC1, *Beauveria* in FC2, and *Botrytis* in FC3 were the biomarkers in their corresponding groups (Supplementary Figure S2D). Ten differential fungal genera were screened using random forest analysis. They were *Plectosphaerella*, *Scedosporiu*, *Chaetomium*, *Paraphaeosphaeria*, *Beauveria*, *Botrytis*, *Talaromyces*, *Alternaria*, *Coniosporium*, and *Ustilaginoidea* (Figure 3B). These differential fungal genera contain all the genus level biomarkers screened by our LEfSe analysis.

### 3.4. Potential Functional Pathways of Rhizosphere Soil Microorganisms during FC Cultivation

The non-redundant genes were functionally annotated based on the KEGG database, and a total of 363 KEGG Level 3 pathways were annotated, of which 323 were annotated by FC1 and 352 were annotated by FC2 and FC3. Ribosome, valine, leucine, and isoleucine biosynthesis, synthesis, and degradation of ketone bodies and lipid acid metabolism pathways contributed the most (more than 2%). Moreover, the relative abundance of genes related to these pathways in the FC2 or FC3 group was greater than that in the FC1 group (Supplementary Table S9).

### 3.5. Metabolomic Analysis of FC Rhizosphere Soil

**Figure 4:**
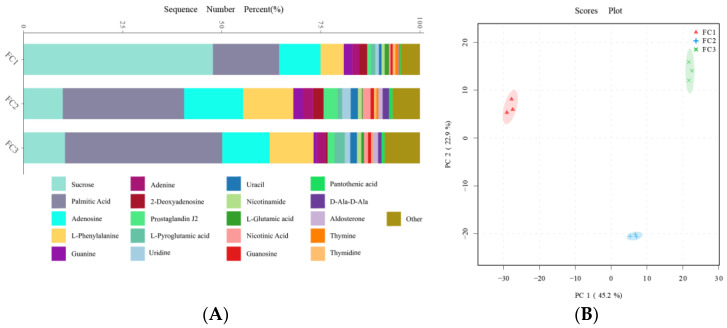
Composition (**A**) and change (**B**) of metabolites in rhizosphere soil during FC cultivation.

**Figure 5:**
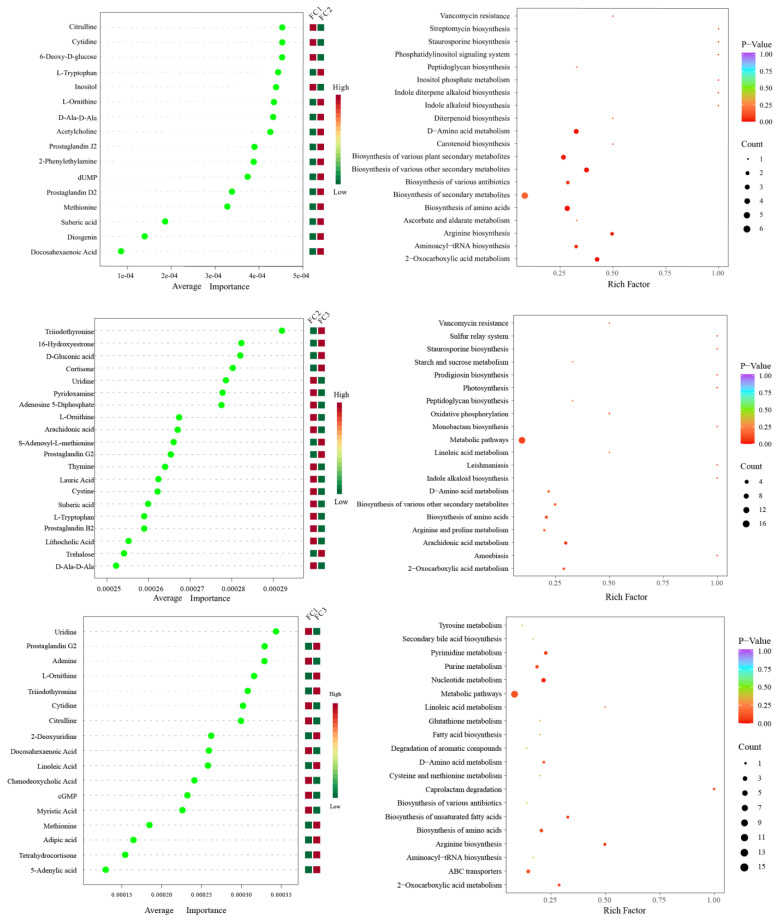
Support vector machine analysis was used to screen differential metabolites between groups, and KEGG enrichment pathway analysis was used for differential metabolites.

After the KEGG database annotation, 103 compounds with biological roles were obtained (Supplementary Table S10). Among them, the relative content of carbohydrates in the rhizosphere soil of FC decreased significantly after more than two years of cultivation, while lipids, peptides, vitamins and cofactors, hormones and transmitters, and steroids showed the opposite trend. Interestingly, the relative content of nuclear acids increased first and then decreased (Supplementary Figure S3). We further analyzed the composition and changes of the compounds with the relative content of the top 20 of them (Figure 4A). The results showed that sucrose was the compound with the highest relative content, and its abundance reached 34.23%, followed by palmitic acid (23.65%), adenosine (11.39%), L-phenylalanine (7.94%), and Guanine (2.00%). In the second and third years of FC cultivation, the relative contents of palmitic acid, adenosine, L-phenylalanine, adenine, and prostaglandin J2 in rhizosphere soil were higher than those in the first year, but the relative contents of sucrose were significantly lower than those in the first year. PCA analysis revealed the similarity and difference of metabolites in the rhizosphere soil of FC during cultivation, and the results showed that each group could be well separated. PC1 explained 45.2% of the variability, and PC2 explained 22.9%. With regard to PC1, FC2 is closer to FC3 than FC2 is to FC1, indicating that the composition and structure of FC2 and FC3 metabolites are more similar (Figure 4B). Support vector machines were used to screen differential metabolites between groups (Figure 5). Sixteen differential metabolites were screened between FC1 and FC2 groups, of which four were significantly downregulated, and 12 were significantly upregulated. Then, these differential metabolites were enriched for metabolic pathways. The results showed that among the top 20 metabolic pathways, they mainly included the biosynthesis of secondary metabolites, D-amino acid metabolism, biosynthesis of various antibiotics, biosynthesis of amino acids, Arginine biosynthesis, Aminoacyl-tRNA biosynthesis, 2-oxocarboxylic acid metabolism, etc. Twenty differential metabolites were screened and compared between FC2 and FC3 groups. Seven were significantly upregulated and thirteen were significantly downregulated. These metabolites were mainly enriched in metabolic pathways, the biosynthesis of amino acids, arachidonic acid metabolism, 2-oxocarboxylic acid metabolism, and D-amino acid metabolism. Similarly, we also screened 17 metabolites with significant differences between FC1 and FC3 groups, with 9 being upregulated and 8 being downregulated. These differential metabolites were enriched in metabolic pathways, pyrimidine metabolism, purine metabolism, nucleotide metabolism, D-amino acid metabolism, biosynthesis of amino acids, ABC transporters, and 2-oxalic acid metabolism.

### 3.6. Correlations between Soil Bacteria, Fungi, and Metabolism

**Figure 6:**
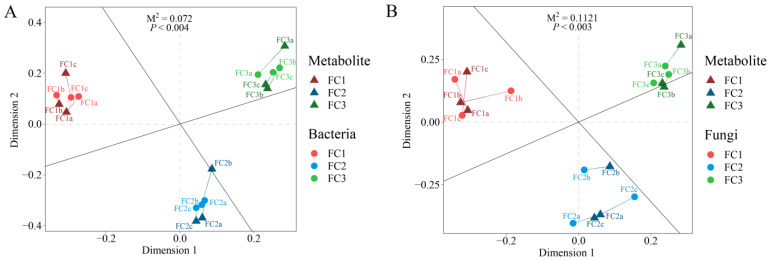
Procrustes analysis was used to evaluate the relationship between bacterial communities and metabolites (**A**) and the relationship between fungal communities and metabolites (**B**).

Procrustes analysis was used to explore the relationship between FC rhizosphere soil microorganisms and metabolites. The results showed that the association between bacteria and metabolites in FC rhizosphere soil (M^2^ = 0.072, *p* < 0.004) was closer than that of fungi and metabolites in FC rhizosphere soil (M^2^ = 0.112, *p* < 0.003) (Figure 6). The correlation heatmap revealed a correlation between differential bacterial genera and differential fungal genera (Supplementary Figure S4). The results showed that the positive correlation between different microbial genera was greater than the negative correlation, among which *Talaromyces* had a significant correlation with almost all bacterial genera, with *Alternaria* having a significant negative correlation with *Roseateles*, *Rhodocytophaga*, *Halopseudomonas*, *Adhaeribacter*, and *Nocardioides*, and a significant positive correlation with *Glycomyces*. *Scedosporium* only showed a significant positive correlation with *Adhaeribacter*, *Nocardioides*, and *Erythrobacter*. *Paraphaeosphaeria* was only significantly positively correlated with *Roseateles* and negatively correlated with *Archangium*. *Botrytis* was significantly positively correlated with *Faecalibacterium* and *Roseateles*, and negatively correlated with *Archangium*. *Ustilaginoidea* has a significant positive correlation with *Faecalibacterium*, *Roseateles*, *Adhaeribacter*, and *Nocardioides*, and a significant negative correlation with *Kaistia*. *Beauveria*, *Coniosporium*, and *Plectosphaerella* had no significant correlation with any of the bacterial genera.

**Figure 7:**
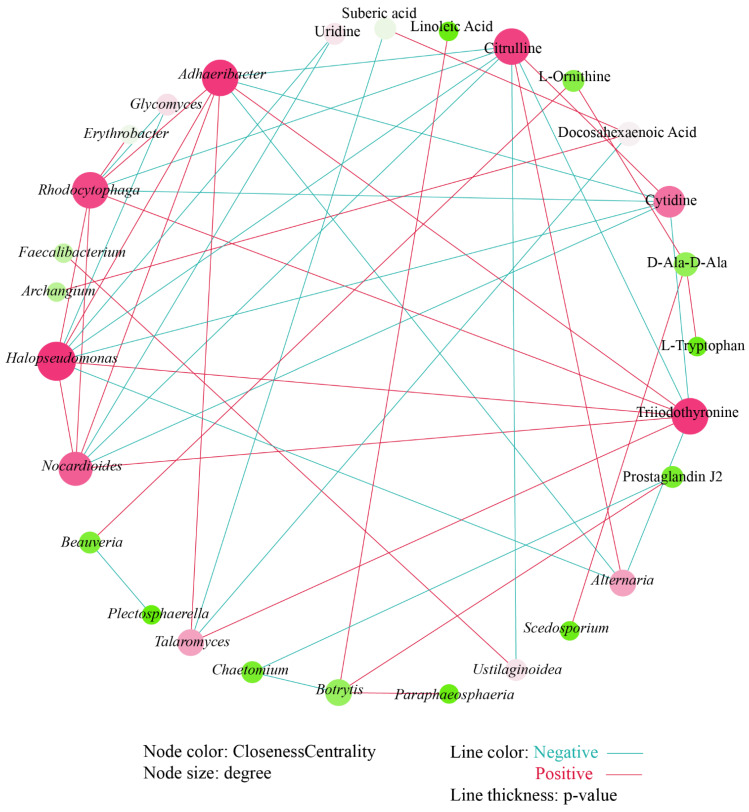
Co-occurrence analysis between differential microbial genera and differential metabolites.

We further conducted a co-occurrence network analysis to explore the relationship between these differential microbial genera and differential metabolites. After deleting the isolated nodes, 11 metabolites, 9 fungal genera, and 8 bacterial genera remained and participate in mapping the correlation network (Figure 7). The correlation network shows that there are more positive correlations (25) than negative correlations (23). *Halopseudomonas* has the most significant relationships with other nodes. Uridine, a differential metabolite, was only significantly negatively correlated with *Halopseudomonas* and *Nocardioides*. There was a significant positive correlation between linoleic acid and *Botrytis*. Prostaglandin J2 has a significant negative correlation with *Chaetomium* and a significant positive correlation with *Botrytis*. Citrulline was negatively correlated with *Adhaeribacter*, *Rhodocytophaga*, *Halopseudomonas*, and *Nocardioides*; negatively correlated with *Ustilaginoidea*; and positively correlated with *Alternaria*. Similarly, cytidine showed a significant negative correlation with the above four bacterial genera. However, triiodothyronine showed a significant positive correlation with the above four bacterial genera and fungal genera *Talaromyces*, and a significant negative correlation with *Alternaria*.

## 4. Discussion

This study was based on metagenomics and metabolomics to preliminarily analyze the composition and changes of the rhizosphere soil microbial community’s structure and rhizosphere soil metabolites during FC cultivation. A combination of ANOSIM and PCoA analyses showed that the microbial intergroup differences were greater than the intragroup differences, and that FC underwent some important changes in the inter-root soil microbial community during cultivation. The diversity and colonization capacity of soil microbial communities in diverse microhabitats not only exert an influence on the growth rate of pathogens but also assume a pivotal role in enhancing plant health. To illustrate, the bacterial community diversity within the rhizosphere soil of diseased *Fritillaria ussuriensis* plants was significantly reduced, accompanied by an increase in pathogen abundance when compared to the state of normal *Fritillaria ussuriensis* plants [32]. The α-diversity of its rhizosphere soil microbial community changed slightly but not significantly during FC cultivation, which was inconsistent with the results of our rhizosphere soil microstudy in *Fritillaria unibracteata*, suggesting that the rhizosphere microbial community of FC was more stable during cultivation, compared to *Fritillaria unibracteata* [33]. This discovery may indicate that FC may be more tolerant to continuous cropping obstacles than *Fritillaria unibracteata*.

Bacteria, being the most plentiful and extensively dispersed category of rhizosphere soil microorganisms, serve as sensitive indicators of alterations transpiring within the rhizosphere’s microecology [34]. *Streptomyces* can significantly promote the inhibition of crop diseases. A recent study showed that *Streptomyces ahygroscopicus* strain 769, which belongs to *Streptomyces*, can significantly reduce the incidence of *Fusarium* wilt of watermelons and increase the biomass of plants [35]. In our study, *Streptomyces* was a microbial genus with significant differences between groups selected via LEfSe analysis. The role of *Streptomyces* in FC continuous cropping is worthy to be investigated further. Among the myriad of bacteria, one particularly noteworthy microorganism is *Mycolicibacterium gilvum*, which falls under the taxonomic classification of the genus *Mycolicibacterium*. The relative abundance of this strain reached 9.56% in rhizosphere soil in the third year of FC cultivation, indicating the prominent role of *Mycolicibacterium gilvum* in the process of FC cultivation. Studies have shown that it has an excellent ability regarding the degradation of polycyclic aromatic hydrocarbons [36], but more research is needed on its interaction with plants. In addition, the relative abundance of *Nocardioides* showed the same trend. Some studies have shown that its abundance in soil with the continuous cropping of tobacco disease is significantly higher than in soil without continuous cropping. However, some studies have also demonstrated that *Nocardioides* has beneficial effects, such as degrading herbicides in soil and secreting actinomycin, which can antagonize the growth of soil pathogenic bacteria [37,38,39]. The relative abundance of *Lysobacter*, known for its ability to decompose deleterious substances and secrete growth-promoting compounds, exhibits a discernible decline upon the cultivation of FC, with a notable disparity observed between its abundance in the rhizosphere of healthy *Fritillaria taipaiensis* and that of diseased *Fritillaria taipaiensis* [40,41,42]. The relative abundance of beneficial bacteria *Pseudomonas* also showed a decreasing trend, which was consistent with the change of *Pseudomonas* in apple rhizosphere soil under continuous cropping, suggesting that we should pay attention to the pests and diseases brought by continuous cropping [43]. Without a disturbance, such bacterial genera are biomarkers that can be predicted via LEfSe analysis. For fungal communities, changes in the relative abundance of several pathogenic fungal genera are noteworthy. For example, the relative abundance of *Cladosporium* was significantly greater in the second and third years of cultivation than in the first year, and it has been suggested that it is the main pathogen causing the apple succession disorder [44]. *Cladosporium cladosporioides*, which causes strawberry blossom blight and Sambucus chinensis leaf blight, is a major member of *Cladosporium* [45,46]. The relative abundance of another pathogenic fungal genus, *Botrytis*, increased significantly in the third year of FC cultivation. Moreover, the model pathogenic fungus, *Botrytis cinerea*, is a major member of aforementioned genus, which can damage host mechanical tissues through the secretion of pathogenic toxins, cell wall-degrading enzymes, and by affecting the expression of pathogenic genes; additionally, it is the major pathogenic fungus of a wide range of fungal plant diseases [47,48]. These changes in pathogenic fungi show the urgent need for attention to be paid to the control of fungal diseases in the later stages of FC cultivation. The relative abundance of *Pseudogymnoascus* as a soil-dominant fungus, which may be involved in the control of pests and diseases—such as infective nematodes and fungi, decomposition of root fragments, and has the role of aiding the uptake of water and nutrients by vegetation—has declined [49].

The research results indicate that the cultivation of FC can cause significant changes in rhizosphere soil metabolites. The relative abundance of sucrose, the main organic matter in the soil, significantly decreased, while the relative abundance of compounds such as Palmitic Acid, Adenosine, and L-Phenylalanine increased compared to FC1 in the FC2 and FC3 groups. Correspondingly, the star and cross metabolism pathway is an important pathway that affects the rhizosphere soil environment of FC (Figure 5). The decrease in soil organic matter will lead to a decrease in the available carbon source for soil bacteria, resulting in a decrease in the relative abundance of the majority of co trophic anaerobic bacteria (Firmicutes and Proteobacteria). Palmitic acid is one of the main root exudates of many plant species, such as wheat [50], sesame [23], walnut [39], etc. Studies have found that palmitic acid can inhibit the reproduction of pathogenic fungi and nematodes, effectively improving the soil environment [51]. In addition, palmitic acid can also promote the growth of watermelon [52], cucumber [53], and tomato [54] plants. However, some studies have shown that root exudates, such as palmitic acid, inhibit potato growth during continuous cropping and can increase the abundance of pathogenic microorganisms (*Fusarium* and *Mortierella*) in the soil, thereby inhibiting plant growth and development [55]. In our study, the relative abundance of *Fusarium* and *Mortierella* seems to show a decreasing trend (Supplementary Table S7), suggesting that palmitic acid is not harmful to FC, but more research is needed to prove it. Among the intergroup differential metabolites screened by a support vector machine, 11 were filtered at a frequency greater than 1, and 4 were all amino acids. Moreover, amino acid metabolism pathways were significantly enriched, indicating that amino acid substances play a crucial role as the main component of root exudates. Empirical investigations have elucidated the pivotal role of amino acids in the context of continuous cropping soil, encompassing vital functions such as soil amelioration, mitigation of pathogenic bacterial populations, and facilitation of plant growth [56,57].

Procrustes analysis was used to assess the relationship between rhizosphere microorganisms and metabolites. Furthermore, as in many studies, FC rhizosphere soil bacteria were more closely related to metabolites than fungi were to metabolites [15,39], showing that bacteria play an important role in shaping plant rhizosphere’s microecology. In the co-occurrence analysis of microorganisms and metabolites, the node sizes of different fungal genera were similar, indicating that these fungi contribute equally to the metabolites of FC rhizosphere soil. Citrulline has the function of catalyzing arginine to generate nitric oxide through nitric oxide synthase, which has the function of slowing down the damage of stress to the plasma membrane and improving antioxidant activity [58]. Citrulline continues to decrease during the cultivation process of FC, and it is negatively correlated with all significantly related bacterial genera, including *Adhaeribacter*, *Rhododytophaga*, *Halopseudomonas*, and *Nocardioides*, suggesting that these bacterial genera may have adverse effects on the growth and development of FC. In addition, the significant positive correlation between Triiodothyronine and microorganisms is also noteworthy, although there are currently no reports of its interaction with soil microorganisms.

## 5. Conclusions

In conclusion, we performed metagenomic sequencing and metabolomic analysis of rhizosphere soil during FC cultivation. The results showed that rhizosphere microorganisms and metabolites changed significantly during FC cultivation. Random forest analysis was used to screen differential microorganisms between groups. The results showed that during FC cultivation, the relative abundance of some beneficial bacteria genera such as *Bradyrhizobium*, *Lysobacter*, and *Pseudomonas* decreased significantly, while the relative abundance of some pathogenic fungi genera such as *Botrytis*, *Talaromyces*, and *Ustilaginoidea* increased significantly. The relative abundance of carbohydrates (especially sucrose) in rhizosphere soil decreased significantly, while the metabolites of lipids and nucleic acids (including amino acids) increased significantly.

## Figures and Tables

**Table 1:** FC rhizosphere soil microbial metagenome sequencing data statistics.

Sample ID	Raw Reads (#)	Raw Base (GB)	%GC	Raw Q20 (%)	Raw Q30 (%)	Clean Reads (#)	Cleaned (%)	Clean Q20 (%)	Clean Q30 (%)
FC1a	23,485,550	7.05	63	95.75	89.71	21,890,302	93.21	97.73	92.72
FC1b	22,148,275	6.64	63	95.76	89.74	20,665,303	93.30	97.73	92.72
FC1c	20,894,601	6.27	62	95.67	89.61	19,299,670	92.37	97.75	92.76
FC2a	24,805,971	7.44	63	95.55	89.39	22,862,774	92.17	97.69	92.65
FC2b	22,057,972	6.62	63	95.64	89.52	20,372,676	92.36	97.72	92.69
FC2c	21,136,778	6.34	63	95.24	88.81	19,629,586	92.87	97.47	92.15
FC3a	26,133,973	7.84	64	95.46	89.24	24,232,350	92.72	97.63	92.51
FC3b	24,475,606	7.34	64	95.51	89.33	22,811,364	93.20	97.63	92.51
FC3c	22,025,034	6.61	64	95.34	88.98	20,330,366	92.31	97.56	92.31

## Data Availability

The data presented in this study are deposited in the NCBI SRA repository, accession number PRJNA1034553.

## Supplementary Materials

The following supporting information can be downloaded at: https://www.mdpi.com/article/10.3390/biology13050334/s1, Table S1: OTU abundance table of bacterial communities from FC rhizosphere soil metagenome sequencing. Table S2: The composition of bacterial communities at the phylum level. Table S3: The composition of bacterial communities at the genus level. Table S4: The composition of bacterial communities at the species level. Table S5: OTU abundance table of fungal communities from FC rhizosphere soil metagenomic sequencing. Table S6: The composition of fungal communities at the phylum level. Table S7: The composition of fungal communities at the genus level. Table S8: The composition of fungal communities at the species level. Table S9: Functional annotation of KEGG Level 3 pathways. Table S10: Compounds with biological functions identified via LC-MS/MS analysis of FC rhizosphere soil. Figure S1: Part (A): dilution curve of bacterial community richness. Part (B): ANOSIM analysis of bacterial community. Part (C): the top 10 bacterial species in the relative abundance of bacterial community. Part (D): LDA score figure of bacterial community LEfSe analysis. Figure S2: Part (A): dilution curve of fungal community richness. Part (B): ANOSIM analysis of fungal community. Part (C): the top 10 fungal species in the relative abundance of fungal community. Part (D): LDA score figure of fungal community LEfSe analysis. Figure S3: Classification histogram of metabolites that play biological functions in FC rhizosphere soil. Figure S4: Correlation heatmap between differential bacteria and fungi.
