# Supplementary figures and images for: Changes in Rhizosphere Soil Microorganisms and Metabolites during the Cultivation of Fritillaria cirrhosa
Source: Biology (Basel). 2024 May 11;13(5):334. doi: 10.3390/biology13050334 (PMC11117757; doi:10.3390/biology13050334)

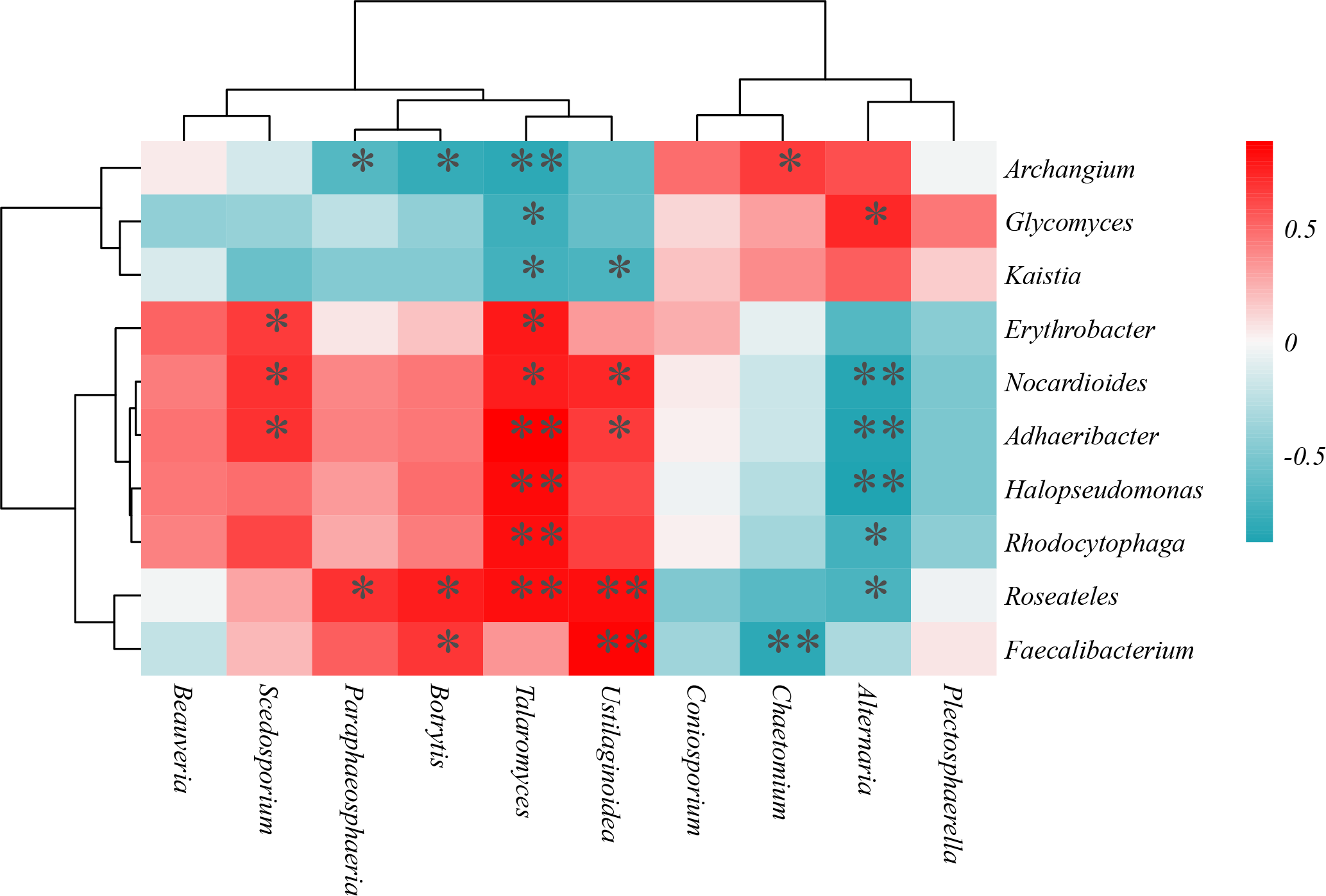

Supplement: Supplementary file 1 [file biology-13-00334-s001.zip › Supplementary Material/Supplementary Figure 4 .tif]

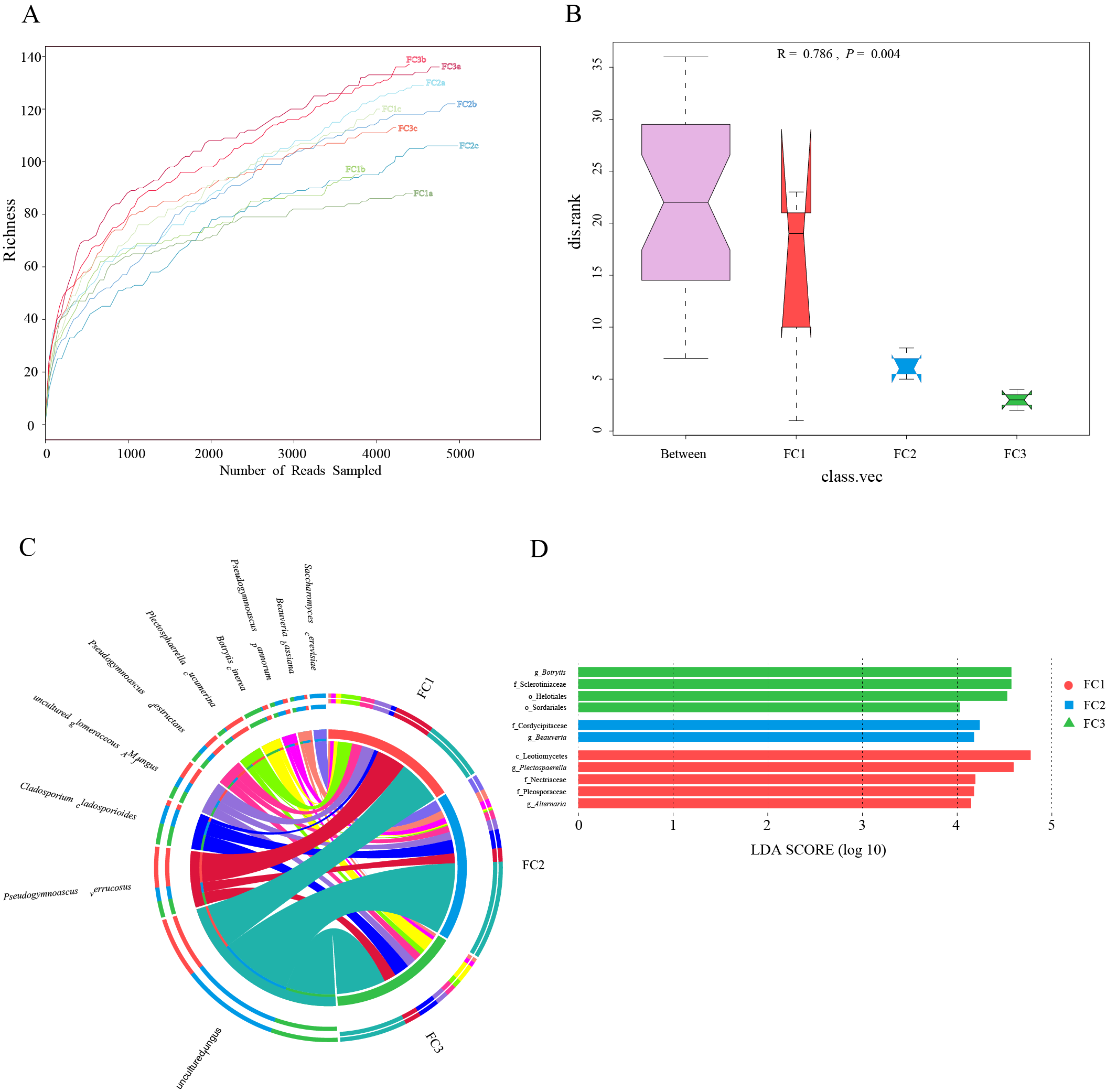

Supplement: Supplementary file 1 [file biology-13-00334-s001.zip › Supplementary Material/Supplementary Figure 2 .tif]

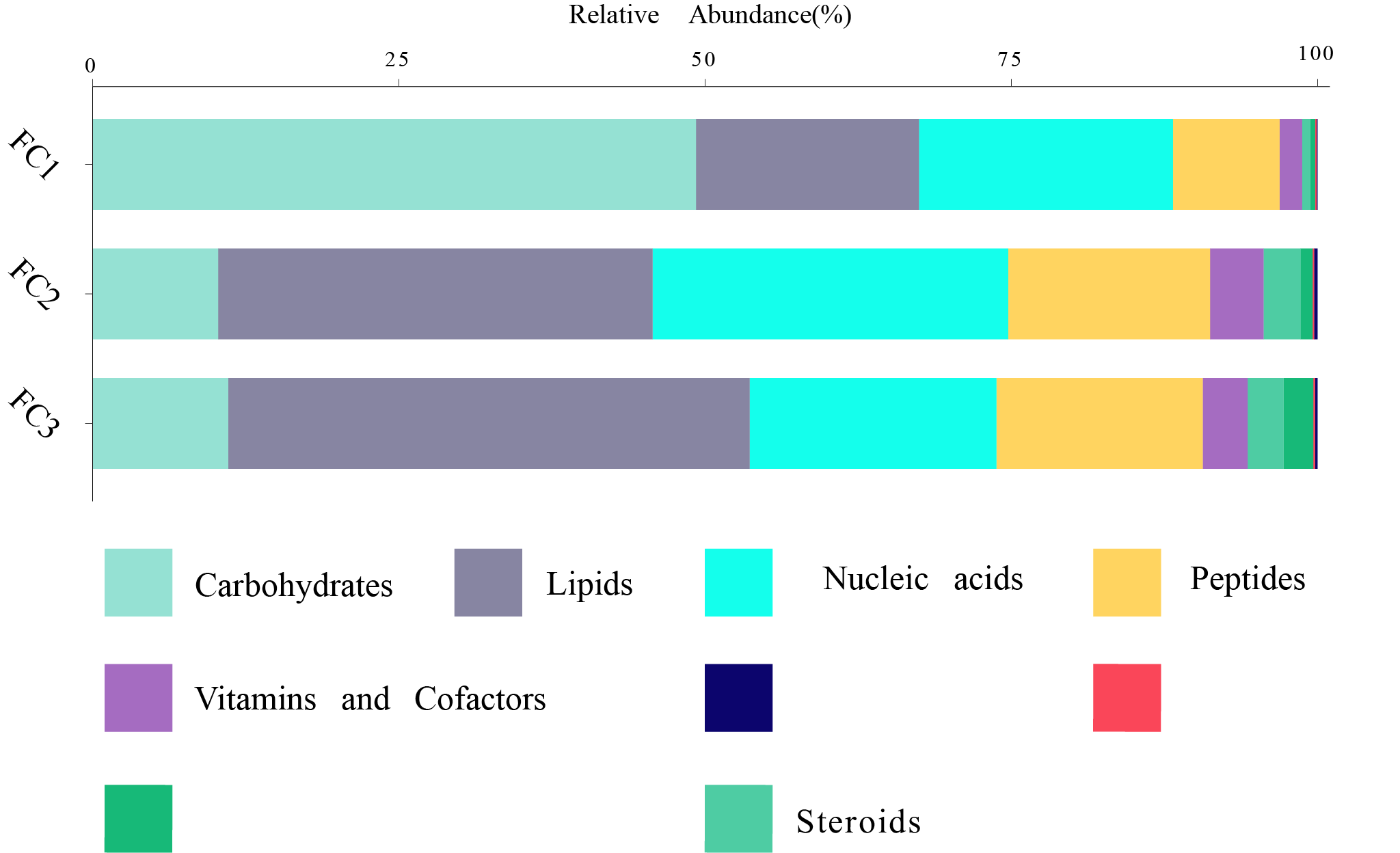

Supplement: Supplementary file 1 [file biology-13-00334-s001.zip › Supplementary Material/Supplementary Figure 3 .tif]

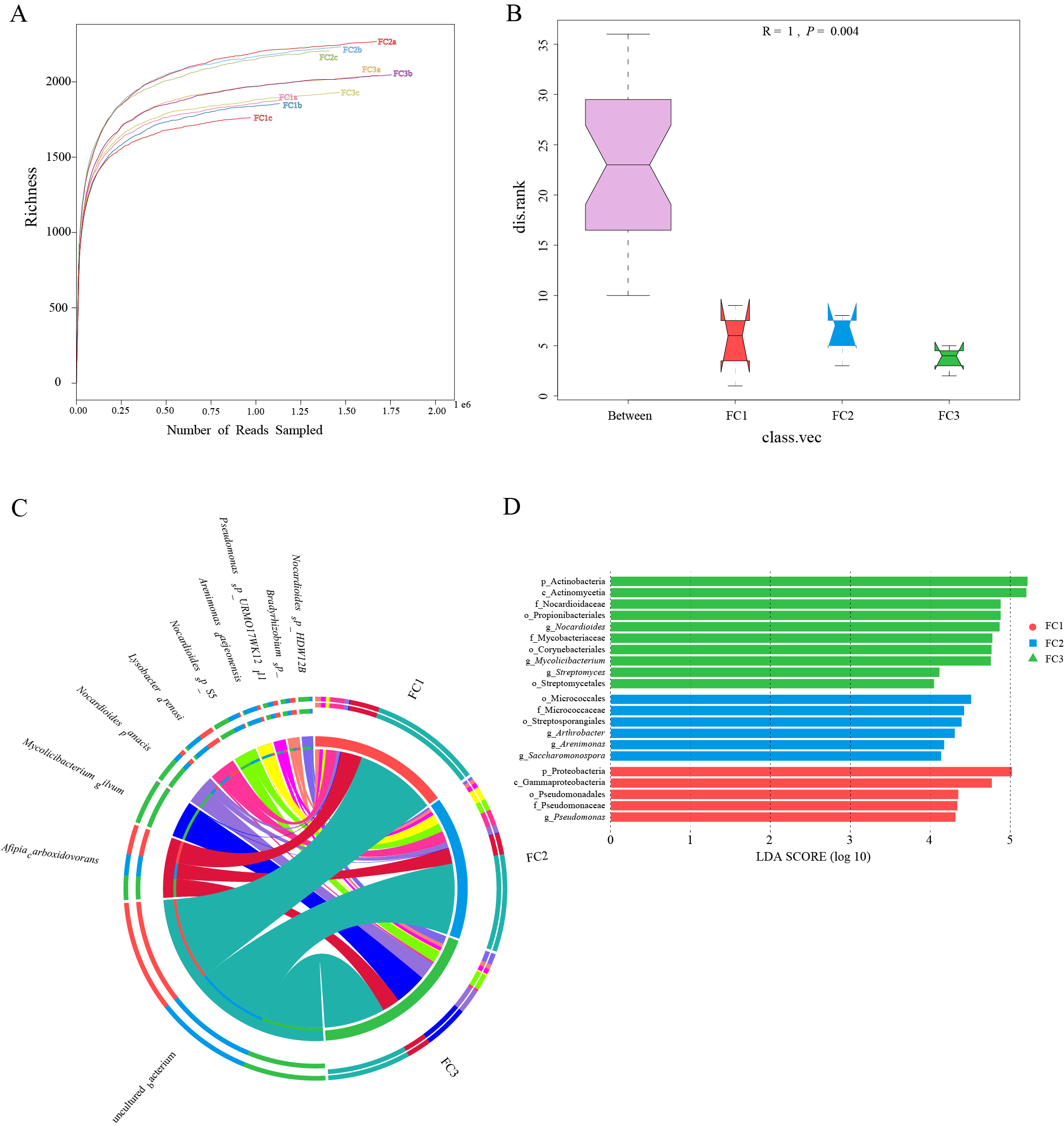

Supplement: Supplementary file 1 [file biology-13-00334-s001.zip › Supplementary Material/Supplementary Figure 1.tif]
